# A Novel Ionic Exchange Membrane Crystallizer to Recover Magnesium Hydroxide from Seawater and Industrial Brines

**DOI:** 10.3390/membranes10110303

**Published:** 2020-10-24

**Authors:** Daniele La Corte, Fabrizio Vassallo, Andrea Cipollina, Marian Turek, Alessandro Tamburini, Giorgio Micale

**Affiliations:** 1 Dipartimento di Ingegneria, Università di Palermo-Viale delle Scienze ed.8, 90128 Palermo, Italy; daniele.lacorte@unipa.it (D.L.C.); fabrizio.vassallo@unipa.it (F.V.); alessandro.tamburini@unipa.it (A.T.); giorgiod.maria.micale@unipa.it (G.M.); 2 Faculty of Chemistry, Department of Inorganic, Analytical Chemistry and Electrochemistry, Silesian University of Technology (SUT)—B. Krzywoustego 6, 44-100 Gliwice, Poland; marian.turek@polsl.pl; 3 ResourSEAs SrL -Viale delle Scienze ed. 16, 90128 Palermo, Italy

**Keywords:** membrane crystallizer, brine valorisation, bittern, wastewater, critical raw material

## Abstract

A novel technology, the ion exchange membrane crystallizer (CrIEM), that combines reactive and membrane crystallization, was investigated in order to recover high purity magnesium hydroxide from multi-component artificial and natural solutions. In particular, in a CrIEM reactor, the presence of an anion exchange membrane (AEM), which separates two-compartment containing a saline solution and an alkaline solution, allows the passage of hydroxyl ions from the alkaline to the saline solution compartment, where crystallization of magnesium hydroxide occurs, yet avoiding a direct mixing between the solutions feeding the reactor. This enables the use of low-cost reactants (e.g., Ca(OH)_2_) without the risk of co-precipitation of by-products and contamination of the final crystals. An experimental campaign was carried out treating two types of feed solution, namely: (1) a waste industrial brine from the Bolesław Śmiały coal mine in Łaziska Górne (Poland) and (2) Mediterranean seawater, collected from the North Sicilian coast (Italy). The CrIEM was tested in a feed and bleed modality in order to operate in a continuous mode. The Mg^2+^ concentration in the feed solutions ranges from 0.7 to 3.2 g/L. Magnesium recovery efficiencies from 89 up to 100% were reached, while magnesium hydroxide purity between 94% and 98.8% was obtained.

## 1. Introduction

The need for raw materials is an essential aspect of the industrial sector of each country. Since the world resources distribution is often uneven, governments are continually searching for new resources. In particular minerals of high value are typically extracted from mines according to a conventional linear industrial approach. Moving towards a more sustainable exploitation of available resources, seawater, and natural and anthropogenic brines can play an important role: as a matter of fact, these practically represent an inexhaustible source of valuable raw materials. They are usually rich in different minerals: Na^+^, Mg^2+^, Ca^2+^, K^+^ are the more abundant, but traces of Li^+^, Rb^+^ and Cs^+^ can be also found [1,2]. Anthropogenic brines can have more variable composition compared to natural brines and seawater, as their salinity is usually much higher and they can contain organic compounds and heavy metals. In most cases, industrial brines are a waste product, which should be suitably disposed of, i.e., properly treated before disposal in order to comply with regional environmental regulations and ecological balance [2].

The most common disposal methods are surface water discharge, deep-well injection, sewer discharge, evaporation ponds and land application [3,4,5,6]. Each method has its advantages and disadvantages in terms of costs and environmental impact and not all of them can be applied for high-salinity solutions. In surface water discharge and deep-well injection, the effluent is discharged directly without particular pre-treatments, thus possibly resulting as harmful to local fauna and flora. The high salinity of the brine may cause hypersaline stress which interrupts ionic homeostasis [7]. Other methods such as sewer discharge [5] and land application [6], cannot be used for brines being excessively salty. Conversely, the evaporation ponds can be used to manage brines with high total dissolved solids (TDS). The ponds consist of lined earthen basins that allow the evaporation of the brines via solar radiation [6]. Notably, none of the methods cited, except for the land application and well-designed evaporation basins, allows the recovery of resources.

Any industrial waste brine treatment process has a cost for the industry. Thus, proposing technologies able to treat brines and recover valuable materials from them is a matter of crucial importance nowadays because it would allow disposal costs to be reduced and a circular economy approach to be implemented. This is why the interest towards brining mining and the valorisation approach has rapidly increased in recent years [4,8,9,10,11,12].

In most cases, mining minerals from brines is carried out by increasing the concentration of salts dissolved up to supersaturation in order to crystallize them. This methodology includes different technologies among which solar evaporation is undoubtedly the most common. It consists of one or more shallow ponds where brine is progressively concentrated until minerals start precipitating and can be harvested. It is basically a very simple and rough example of fractionated crystallization. Solar evaporation has been known since ancient times, when it was typically adopted to recover salt from seawater. According to this methodology, only salts present in high concentration can be crystallized [8].

Minerals in low concentration can be recovered by the adsorption/desorption method. Several inorganic or organic compounds can bind minerals selectively. Li, U, Sr and Rb are the elements whose recovery has been more studied due to their economic importance and rarity. They can be recovered from seawater or industrial brine using compounds such as MnO_2_, calcium alginate or cobalt hexacyanoferrate that have a good adsorption ability [13,14,15]. According to the most common procedure, these compounds adsorb the elements from the brine solution and then the elements are desorbed in a small amount of solvent and finally crystallized (usually via evaporation or reactive crystallization). Therefore, the adsorption/desorption method is a low-cost process, but a good selectivity has not been reached yet and multiple adsorption steps are needed, thus making the recovery of high-purity products really complex and expensive, especially when high salty brines are dealt with [13,14,15].

Membrane-based technologies are also adopted to recovery raw materials from brines. In particular, electrodialysis (ED) and membrane distillation crystallization (MDC) [16,17,18] are those most studied. Both technologies take advantage of the selective properties of the membrane to change the composition of a solution, typically concentrating a stream in salts, thus making their recovery easier. ED makes use of suitably tailored ion exchange membranes (IEM) along with an externally applied electric field to promote the migration of ions. More precisely, IEMs being selective towards monovalent ions are used in order to separate mono from divalent ions, thereby allowing them to be crystallized separately by a conventional method (evaporation or chemical precipitation). The high cost of electric energy and scaling issues are the main technology limitations [19]. Conversely, MDC employs hydrophobic microporous membranes and thermal energy in order to achieve supersaturation conditions. Curcio et al. [20] were the first to name this MDC technology as “membrane crystallization”, although MDC is actually used to concentrate the solution, while massive crystallization is carried out in a conventional crystallizer following the MDC unit. This is an innovative process deriving from membrane distillation in which the solvent is evaporated to increase salt concentration. The two sides of the membrane have different temperatures. The solvent evaporates on the warm side and condenses on the cold side. The hydrophobic nature of the membrane allows the vapour to pass through itself and prevents the condensed water from coming back. It was proven that this technology can guarantee a tight control of supersaturation and a consequent control of crystal size distribution and morphology [21]. The crystallization is performed in a separate vessel in order to minimize membrane fouling. Clearly, MDC can allow the recovery only of the salts exhibiting the highest concentration in the solution. Another special application of membrane crystallization relies on the use of solvent/antisolvent. More precisely, a solution to be crystallized feeds the channel of the membrane module, conversely, the anti-solvent is used to feed the other channel. The latter is gradually evaporated and passes through the membrane. Once the anti-solvent has reached the crystallizing solution, the solute solubility decreases up to crystallization [21,22].

Reactive crystallization is a valid alternative to recover substances from saline solutions. It is particularly suitable to obtain sparingly soluble or insoluble compounds that, reacting with an appropriate reactant, may encounter a phase separation. This method has been effectively used in the separation and recovery of different ions such as: NH_4_^+^ and PO_3_^2−^ ions, heavy metal ions and alkaline earth metal (calcium and magnesium) ions [23,24,25,26]. Phosphates can be recovered with magnesium as struvite, a crystal of magnesium ammonium phosphate ((NH_4_)MgPO_4_·6(H_2_O)) widely used as fertilizer. Sulphide and hydroxyl ions are suited to react with heavy metals to form a precipitate, which can be easily separated from the original solution. It is often important to remove them from effluents and brines before disposal to avoid biological accumulation and toxicity.

Many studies reported the possibility of recovering magnesium, in the hydroxide form, from a saline solution by reactive crystallization [27,28,29,30,31,32], via a precipitation process driven by the addition of an alkaline reactant:

(1) $$Mg_{\left(aq\right)}^{2+}+2OH_{\left(aq\right)}^{-}=Mg{\left(OH\right)}_{2\;\left(s\right)}↓$$

Magnesium hydroxide has a vast market and can be sold as a final product for pharmaceutical, flame-retardant and environmental protection purposes depending on its purity, morphology and size distribution. Also, it is an intermediate for the production of other magnesium compounds (magnesium metal and magnesium oxide) [33].

Many studies proved the feasibility to recover magnesium from anthropogenic brine, natural brine and seawater. Lime, slaked lime, ammonia and sodium hydroxide are the mostly used reactants [27,28,29,30,31,32]. Lime and slaked lime are the cheapest reactants used in different processes to recover magnesium from saline solution. For example, Dow Chemical Company patented in 1943 [34] a process to recover magnesium from seawater by precipitation with lime. In this case, the production of magnesium hydroxide is an intermediate aimed at producing metallic magnesium. Generally, lime contains impurities such as silicon, iron, aluminium and other insoluble compounds and further purification steps are needed to obtain high-purity products [35].

In recent years, Mohammad et al. [36] and Dong et al. [37] proved the feasibility to recover magnesium hydroxide via the addition of ammonium hydroxide. They used two different artificial brines originating from a desalination plant. The main issue with this methodology is the low conversion percentage. In fact, ammonium hydroxide reacting with magnesium is transformed into ammonium forming the ammonia buffer solution. Therefore, an excess of ammonium hydroxide must be added to increase the pH and achieve a good conversion rate. An optimal NH_3_/Mg molar ratio of 4.4 was found by Mohammad et al. [36]; conversely, Dong et al. [37] reported a NH_3_/Mg ratio of 6.0 and a product purity of 93.5%. This over-stoichiometric amount of reactant clearly implies a significant increase of operative cost. Moreover, the exhausted solution still contains a large amount of unreacted ammonium hydroxide which is a toxic compound and whose disposal is regulated by strict environmental laws, thus further increasing the cost of the whole recovery process and reducing its economic potential.

Cipollina et al. [32] demonstrated the recovery of magnesium from exhausted brines produced by the saltworks of Trapani (Italy). This brine is particularly rich in magnesium ions (about 20–30 times more than the seawater), which were recovered in the form of high-purity magnesium hydroxide by adding NaOH to the brine. A continuous stirred tank reactor (CSTR) was used to perform the reactive crystallization process and purity of the Mg(OH)_2_ crystals was found to range from 98% to 100%.

In reactive crystallization, reactants are mixed together. Thus, high-purity reactants are needed for this process in order not to contaminate the final product, but operating costs increase accordingly. In addition, being seawater and brines composed of many ions, choosing the most suitable alkaline reactant is crucial to guarantee a high product purity: in the worst cases, undesired massive co-precipitations may occur [28].

The above literature findings suggest the need for a new technology able to guarantee an addition of OH^−^ ions to the saline solution without the constrain of employing expensive alkaline solutions [22,38,39]. To this purpose, a novel ion exchange membrane crystallizer (CrIEM) was developed and patented [40] by our research group, which allows the passage of ions of interest (i.e., OH^−^ ions) for crystallization, without the need for the direct mixing between the two solutions, thus deleting any co-precipitation issue. This also allows low-cost and purity reactants or alkaline industrial waste to be used without the risk of decreasing the final product purity.

The present work aims at demonstrating that this novel technology is suitable to recover high-purity magnesium hydroxide from multi-component solutions by a reactive membrane crystallization driven by a cheap alkaline reactant. To this purpose, an experimental campaign was carried out to extract Mg(OH)_2_ from two different saline solutions, the one natural, the other industrial: (1) a waste industrial brine from the Bolesław Śmiały coal mine in Łaziska Górne (Poland) and (2) Mediterranean seawater, collected from the North Sicilian coast (Italy).

## 2. The Ion Exchange Membrane Crystallizer (CrIEM) Technology

**Figure 1:**
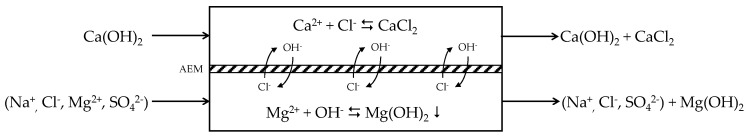
Process scheme of the ion exchange membrane crystallizer (CrIEM) technology for the precipitation of Mg(OH)_2_ from Mg-rich brines.

The CrIEM is a novel crystallization technology based on the use of ion-exchange membranes, patented by Cipollina et.al. in 2015 [40]. It allows reactive crystallization to be performed for the separation of valuable species (e.g., Mg from brines) with large flexibility in the choice of reactants. The CrIEM consists of an ionic exchange membrane separating two different solutions allowing the controlled transport of species (see Figure 1). In particular, an anionic exchange membrane is employed allowing the anions present in the two solutions to migrate from one channel to other one, while rejecting the cations, according to the Donnan exclusion mechanism [41]. From this perspective, it is essential to use an appropriate membrane in order to finely control the passage of species and the subsequent reactive crystallization process.

In our application, the membrane allows the passage of hydroxyl ions from an alkaline solution to the Mg-rich brine compartment, where the crystallization of magnesium hydroxide occurs, while chloride ions move in the opposite direction to comply with electroneutrality. The driving force for the movement of ions is an electro-chemical potential difference between the two channels. This originates from the difference in concentration and is accompanied also by the formation of Donnan potential at the solution/membrane interface.

Since magnesium hydroxide has a very low solubility (Ksp 5.61·10^−12^), the passage of hydroxyl ions lead to the increase of pH and, above 9, the supersaturation is quickly reached so that magnesium ions (Mg^2+^) instantly react with available hydroxide ions and precipitate in the form of Mg(OH)_2_. It is worth noting that while the hydroxyl ions react and precipitate, thus maintaining a low concentration in the brine compartment, chlorides continuously move from the brine to alkaline compartment, thus providing an important additional driving force for the Donnan transport mechanism (Figure 1). Due to the membrane, the alkaline solution does not mix with the brine, thus avoiding any undesired coprecipitation of species other than Mg(OH)_2_ (e.g., calcium sulphates and carbonates).

The main innovative features of CrIEM technology are: (1) the possibility of using a low-cost reactant being unsuitable for conventional reactive crystallization processes (e.g., due to the co-precipitation of undesired products); (2) the opportunity to reduce the environmental impact by selecting the best-performing and less-polluting reactant; (3) the absence of moving parts, which reduces the risk of mechanical failure; (4) modularity and flexibility.

## 3. Experimental Set-Up, Materials and Methodology

### 3.1. Description of the Experimental Set-Up

**Figure 2:**
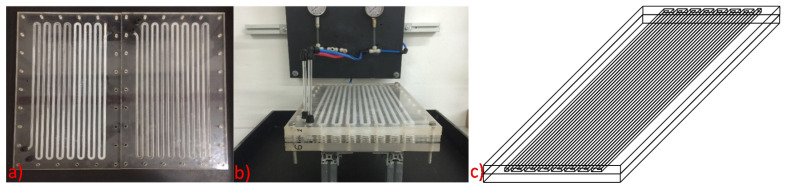
CrIEM reactor, (**a**) disassembled; (**b**) assembled; (**c**) 3D scheme.

The laboratory CrIEM unit adopted in the present work consists of two Plexiglas plates, each one carved with a semi-circular zig-zag shaped channel (Figure 2). This material was chosen in order to allow for a visual inspection of the system during operation. The plate sizes were 535 mm × 325 mm × 20 mm, while the channels diameter was 8.1mm. The total length of the circuit was 7180 mm with a total volume of 187 mL per channel.

**Figure 3:**
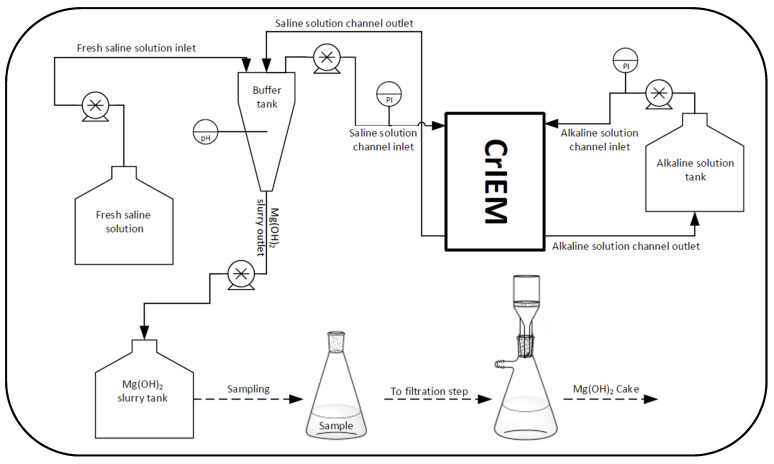
Sketch of the experimental set-up.

The experimental test-rig employed for the experimental campaign is reported in Figure 3. Two hose connectors were used to feed the solutions into the CrIEM device: only polymeric material was adopted to avoid corrosion phenomena caused by the high salinity of solutions and by the alkaline stream due to the high pH. A flat-sheet Fujifilm type 10 anion exchange membrane (AEM) was inserted between the plates, resulting in a net transfer area of about 585 cm^2^ (the characteristics of the membrane are listed in Table 1).

The alkaline solution channel was connected to a storage tank, while the saline channel was connected to a buffer tank. The buffer tank consisted of a cylinder with a conical frustum shape bottom, a total volume of 2750 mL and 47 mm of radius for the cylindrical part. A flexible hose fitting was located at the bottom part of the buffer tank so that the Mg(OH)_2_ –rich suspension could be drained and stored in a separate tank. A pH meter was located within the buffer tank to check the crystallization progress. Furthermore, the upper part allowed the placement of different tubes corresponding to one outlet and two inlets. One inlet and one outlet were relevant to the saline solution exiting from and entering into the CrIEM reactor, respectively; the other inlet allowed the fresh saline solution make-up to the test-rig.

Two pressure gauges were positioned at the inlet of the two channels of the CrIEM in order to control the pressure drops during the process. Four peristaltic pumps (Seko Kronos 50) were used for the movement of all liquids. Two pumps were used to recirculate saline and alkaline solution in the CrIEM from the relevant buffer tanks, while the other two pumps were used to feed the brine to its buffer tank and to drain it, respectively. The feed and bleed arrangement, realised by using the buffer tank as a collector/mixer of reactor-outlet and make-up feed, allowed for a continuous operation mode looking at the brine stream, while a batch circulation was adopted for the alkaline solution in the buffer tank. The Mg(OH)_2_–rich slurry stored in the tank (next to the buffer) was discontinuously sampled (after a suspension volume of about 1 litre was reached in the tank) and then filtered in a laboratory vacuum filtration system to recover the magnesium hydroxide as a solid cake.

### 3.2. Materials and Experimental Procedures

Calcium hydroxide purchased from Sigma-Aldrich (purity >96%) was chosen as alkaline reactant. It is a cheap reactant and sparingly soluble inorganic compound, generally employed in the form of suspension. Using this reactant with traditional crystallization methods, where direct mixing between reactants occur, generally provides a low-purity magnesium hydroxide [35]. Therefore, Ca(OH)_2_ was considered the most suitable to compare the CrIEM performance with that of traditional crystallization methods. Moreover, the adopted Ca(OH)_2_ suspensions could also be regarded as an attempt to mimic typical alkaline industrial wastes [43], which could be used to further promote the circular approach of the proposed technology. In all experiments, a volume of 5 litres of suspension was adopted, with a slurry concentration of 10 g/L of Ca(OH)_2_, periodically substituted in the long-run feed and bleed tests.

The experimental campaign is composed of six different tests listed in Table 2 and briefly described below. Note that each test is identified by a code reported in the second coloumn of the table.

In tests 1 to 4, an industrial brine deriving from the Bolesław Śmiały coal mine in Łaziska Górne (Poland) was employed. The original brine was preliminarily pre-treated by Silesian University of Technology (SUT) personnel at Bolesław Śmiały coal mine premises: after a decarbonization step for removal of soluble CO_2_, carbonates and bicarbonates, the brine was processed in an ultrafiltration stage in order to remove completely sub-micronic coal particles. The resulting brine (TDS ~20 g/L) was fed to a nanofiltration (NF) unit splitting the solution into two-streams, one NaCl-rich permeate and a second magnesium-calcium-rich retentate (TDS above 30 g/L) [44]. The latter stream was used in the present experimental campaign (Tests 1 to 4) to recover Mg(OH)_2_. In particular, tests 1 to 3 referred to three different samples (i.e., different operating days) of the real brine exiting from the NF unit. Test 4 brine resulted from the artificial addition of magnesium chloride hexahydrate (>99%, Chem-Lab) to the brine of test 2 in order to increase the amount of dissolved Mg from 0.77 to 3.2 g/L (trying to mimic the amount of Mg contained in a typical reverse osmosis brine). Tests 5 and 6 were carried out with Mediterranean seawater as saline feed solution (sampling site: Mondello, Italy). Seawater samples were filtered with a 1 μm cartridge filter before the experiments.

In all tests a fixed volume of brine was processed (values are also reported in Table 2).

### 3.3. Methodology

The experimental procedure to recover magnesium from saline solution was composed of two phases: (i) a start-up phase where the system was operated in batch conditions starting from a pristine brine volume, (ii) a continuous operation phase where the brine loop was operated under feed and bleed mode starting from the reacted brine volume.

**Figure 4:**
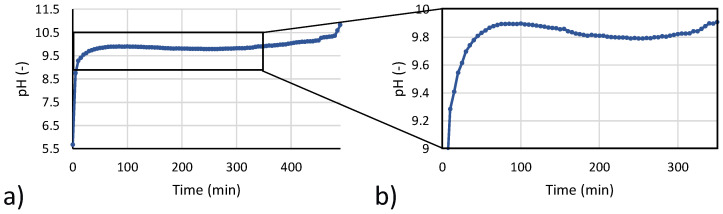
(**a**) pH versus time trend during the start-up phase 4; (**b**) zoom of initial pH vs. time trend.

In the start-up step, the saline solution was recirculated from buffer tank to the CrIEM until all magnesium ions present in solution were converted into Mg(OH)_2_. pH values in the brine buffer tank were continuously monitored by means of a digital pH meter (WTW pH-Cond 3320/SenTix^®^ 41, WTW, Munich, Germany). As shown in Figure 4a, once the test starts, pH rapidly increases until a value around 9.8. Above this value, Mg(OH)_2_ starts reacting massively with hydroxyl ions and precipitating. The precipitation consumes OH^-^ ions in solution, while a continuous passage of OH- through the membrane results in a stabilized value of the pH. When the amount of Mg ions is significantly reduced, pH starts slightly increasing up to around 10.5, when all Mg ions have reacted and pH trend starts exhibiting a sharp increase. A visual observation of the transparent reactor during experiments indicated the solution was clear up to a pH around 9.9, thus suggesting that a massive precipitation had not started yet. The slight drop of pH shown in Figure 4b occurs exactly when the solution starts becoming cloudy due to Mg(OH)_2_ precipitation from a metastable slightly supersaturated solution. The incipient formation of crystals leads to an increase in crystallization rates and a slight reduction in the pH, which then increases again until the equilibrium value.

Once pH above 10.5 was reached and all magnesium had reacted, at least 12 h had to pass before starting the feed and bleed step. This was necessary in order to wait for the settling of magnesium hydroxide in the feed buffer tank and thus improve the bleeding of the slurry with the make-up brine, still being able to continuously remove a dense settled slurry product from the conic section on the bottom of the buffer tank.

After this step, the continuous feed and bleed operation started, where make-up brine was fed to the buffer tank and the magnesium hydroxide slurry was drained simultaneously (see Figure 3).

The two inlets and the outlet of the buffer tank were accurately positioned in order to create a mixing zone in the upper part and a quiet volume in the bottom to promote the settling of particles. In fact, the make-up brine inlet was located near the surface, while the recirculated outlet from the CrIEM was injected peripherally at the beginning of the conic section. The brine outlet feeding the CrIEM unit was positioned near the surface, below the make-up inlet, where the lowest particles concentration could be encountered. Finally, the dense slurry with the settled crystals was withdrawn through one peristaltic pump connected with the bottom of the buffer tank and collected in an Erlenmeyer flask.

The product slurry was sampled and then filtered by means of a laboratory vacuum filtration system. The obtained cake was flushed with distilled water to remove the trapped saline solution, then dried in an oven at 120 °C for at least 8 h and subsequently analysed. Conversely, the filtered solution was analysed in order to calculate the conversion efficiency.

Membrane fouling was preliminarily checked after a test (i.e., Test 2) by disassembling the unit: (i) a thin layer of magnesium hydroxide attached on the membrane was found, (ii) no fouling was observed on the alkaline side. This layer was found not to affect the experiments (e.g., Tests 5 and 6 were run consecutively without disassembling and cleaning the unit and similar conversion times were found).

Moreover, membrane stability within the operating pH was proven by observing no variation in process performance in all tests conducted.

However, future studies and analyses will be specifically devoted to investigating fouling phenomenon (at different operating conditions) and membranes stability in long-run operation, and their long-term influence on the CrIEM performance.

### 3.4. Analytical Procedures and Definition of Performance Parameters

The ionic composition of liquid and solid samples was determined by ion chromatography (IC) Metrohm 882 Compact IC plus (Metrohm AG, Herisau, Switzerland) and cation exchange column Metrosep C4-250/4.0 (Metrohm AG, Herisau, Switzerland). In order to prepare the sample for IC analysis, the filtrate was properly diluted into Milli-Q water without further pre-treatment. Conversely, 100 mg of dried solid was first dissolved in a stoichiometric amount of HCl (TraceSELECT^TM^, Fluka, Charolotte, NC, USA) and subsequently diluted in 1000 mL of Milli-Q water.

Analytical measurements allowed us to calculate the two main performance parameters investigated in the present work: (i) magnesium recovery efficiency; (ii) calcium loss and (iii) magnesium hydroxide purity.

Magnesium recovery efficiency represents the % of magnesium harvested from the feed brine in the form of magnesium hydroxide. It can be calculated from the concentration of the filtered solution sampled during the test according to the following equation:

(2) $$\frac{\left(C_{Mg-in}-C_{Mg-out}\right)}{C_{Mg-in}}\;\times 100\;\left[\%\right]$$

where *C_Mg-in_* is the inlet saline solution Mg concentration, while *C_Mg-out_* is the Mg concentration in the sampled outlet solution, after filtration for solids separation.

Calcium loss represents the % of calcium co-precipitated from the feed saline solution during the process. It can be calculated, similarly as the magnesium recovery efficiency, from the concentration of the filtered solution sampled during the test according to the following equation:

(3) $$\frac{\left(C_{Ca-in}-C_{Ca-out}\right)}{C_{Ca-in}}\times 100\;\;\left[\%\right]$$

where *C_Ca-in_* is the inlet saline solution Ca concentration, while *C_Ca-out_* is the Ca concentration in the sampled outlet solution, after filtration for solids separation.

Magnesium hydroxide purity is defined as the % of magnesium present among all main cations detected by the IC analysis in the sampled, filtered and washed solid. Thus, it can be calculated as:

(4) $$\frac{C_{Mg}^{s}}{\sum^{\;}C_{i}^{s}}\;\times 100\;\left[\%\right]$$

where $C_{Mg}^{s}$ represents the concentration of magnesium detected by ion chromatography from the dissolved solid sample, while $C_{i}^{s}$ represent the concentration of any cation detected by ion chromatography.

## 4. Results and Discussion

### 4.1. Analysis of Mg^2+^ Recovery in Continuous Feed and Bleed Tests

**Figure 5:**
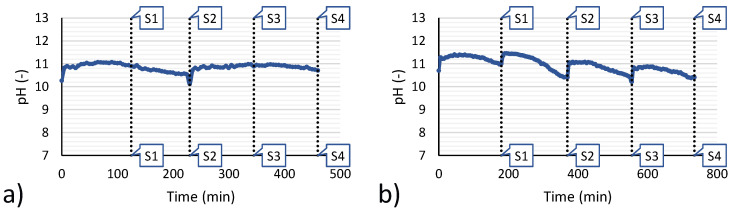
Time variation of pH during the feed & bleed tests. (**a**) test 3; (**b**) test 6. Vertical dotted lines indicate the time at which samples for analysis were taken (samples number is used as reference in Figure 6).

As a first observation of experimental results, it is worth analysing the behaviour of the system in terms of pH during the whole duration of a feed and bleed continuous precipitation test. As an example, Figure 5 reports the pH trends for the reference tests 3 and 6.

After all the magnesium was reacted, the pH can change rapidly because no buffering agents are present. Therefore, the inlet and outlet flow rate of make-up brine and magnesium hydroxide slurry was accurately chosen. With 9 mL/min (test 3) and 5 mL/min (test 6), the optimal operating flow rate was obtained, which guarantees the pH will remain within a range 10.4 to 11.7. In fact, below pH 10.4 too many magnesium ions remain unreacted, decreasing the recovery efficiency. Conversely, above pH 11.7 the precipitation of Ca(OH)_2_ can start. Thus, operating in this range of pH represents a good trade-off to allow for high values of magnesium recovery efficiency and low values of calcium loss, the latter being the main factor reducing the solid purity.

Focussing again on the pH trend of tests 3 and 6, small differences in behaviour can be observed, generated by the manual adjustment of flowrates but also amplified by the fact that the test n.6 had a double duration and the sampling intervals were also doubled, which generated a large variation in the pH due to the consumption of alkaline reactant.

**Figure 6:**
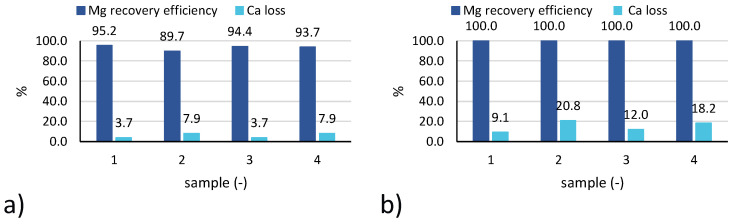
Magnesium recovery efficiency and calcium loss for 4 samples collected during: (**a**) test 3; (**b**) test 6.

The Mg recovery efficiency (Figure 6a) is above 89% in all cases, with slightly larger values achieved for test n.6 (practically 100% for all samples).

Concerning Ca losses (Figure 6b), values recorded for test 6 were higher than for test 3, likely due to the lower initial concentration of calcium in the feed brine, although similar amounts of calcium were precipitated during the test (as confirmed by the similar values of purity reported in Figure 8).

**Figure 7:**
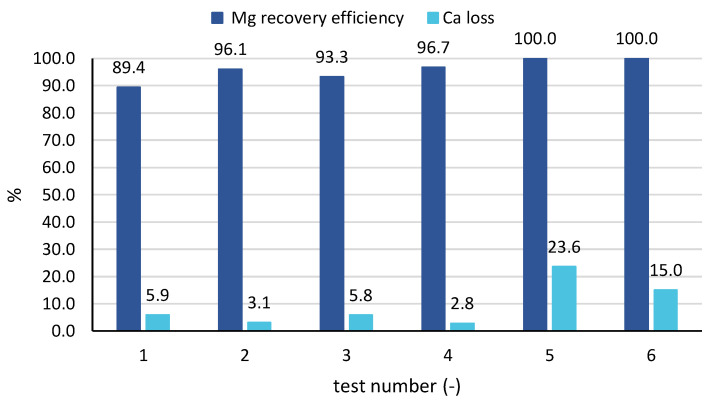
Averaged values of magnesium recovery efficiency and calcium loss in all tests 1–6.

When looking at the averaged values of performance parameters for all samples of each (Figure 7), results confirm the findings analysed above for tests 3 and 6. In particular, magnesium recovery efficiency started from values around 90% up to 96–97% for the tests with real brines, while reaching values around 100% for the tests with seawater. Conversely, the percentage of calcium precipitated with the solid was higher for the test carried out with seawater (15% and 24%) than for those carried out with the industrial brine (3–6%). The cause can be twofold: (1) with industrial brines pH values are better controlled and are kept under a lower limit; (2) calcium concentration in seawater is lower than in the industrial brine, thus leading to a larger percentage of precipitated calcium salts.

### 4.2. Analysis of Mg Purity in the Solid Samples Obtained in Continuous Feed and Bleed Tests

Solid samples processed and analysed according to the aforementioned procedure have been characterised in terms of magnesium purity (against the presence of other cations, see Equation (4)).

**Figure 8:**
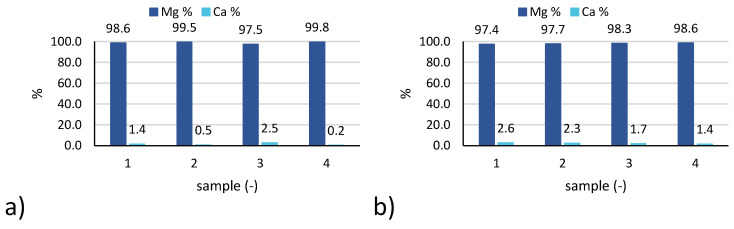
Solid purity expressed as percentage of magnesium and calcium ions in the 4 solid samples collected during: (**a**) test 3; (**b**) test 6.

Also in this case, the results relevant to 4 samples of reference tests 3 and 6 are reported in Figure 8. 

In particular, the percentage of magnesium and calcium in the solid samples are reported, being the concentration of other cations (namely, Na^+^, K^+^, NH_4_^+^) below the IC detection limit.

**Figure 9:**
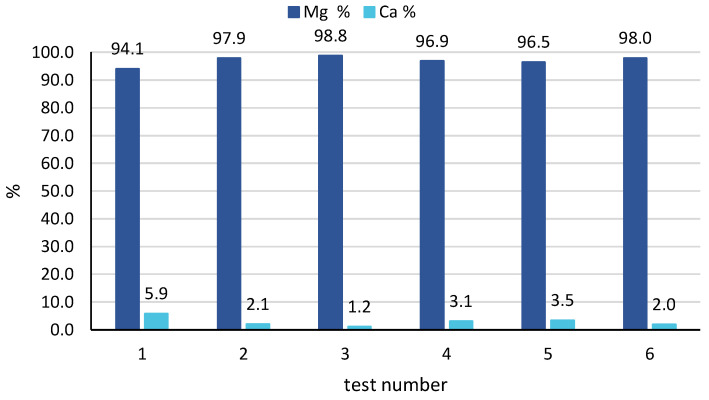
Solid purity expressed as percentage of magnesium and calcium ions, as an average of all samples collected in all tests 1–6.

In all cases, a very high purity magnesium hydroxide was produced, with a percentage of magnesium higher than 97%, exceeding 99% in many samples collected. As said above, the only impurity was related to the presence of calcium salts, with a Ca % typically below 3%. No significant variation in solid composition was observed in the 4 samples, thus indicating a fairly stable behaviour of the CrIEM reactor throughout the 8 to 12 h continuous tests. Looking at the averaged results for all tests performed, very good results were confirmed, as reported in Figure 9. In fact, a high solid purity was obtained in all tests, with a minimum value of 94% observed for test 1, while values around or above 97% were registered for all other tests. The best performance, in terms of average solid purity, was achieved by tests 3 and 6 with average values of 99% and 98%, respectively. This indicates that, by properly controlling and optimising the operating conditions of the CrIEM unit, a high-purity product can be obtained both from an industrial brine (e.g., the coal mine brine) and from a natural saline solution such as seawater.

## 5. Conclusions

In the present work, a novel ionic exchange membrane crystallizer was presented as an innovative technology for the recovery of magnesium from different saline solutions. The use of an ion exchange membrane in the CrIEM guaranteed high recovery rates, with a high purity of the magnesium hydroxide produced, notwithstanding the use of very cheap alkaline reactant, which would lead to very low quality of crystals in conventional CSTR reactive crystallizers.

Adopting a feed and bleed configuration for continuous operation of the system ensured for all investigated conditions a high magnesium recovery efficiency (between 90% and 100%) and a solid purity in magnesium around or above 97% in all cases but one which achieved a purity of 94%. The co-precipitation of calcium ions still represents the main limitation to the achievement of 100% purity in the solid product.

Future activities will aim at the scale-up strategies and long-term stability of the system, also considering other types of feed solutions. Moreover, as technological developments and spin-off ideas, the use of cleaning ball strategies to minimise fouling in continuous operation and the adoption of active ED with bipolar membranes for the in situ generation of alkaline reactant will be considered.

## Figures and Tables

**Table 1:** Properties of the Fujifilm anion exchange membrane (AEM) membrane type 10 [42].

Item	Unit	Specification
Selectivity	%	95–98
Water Permeability	mL/hr/bar/m^2^	8
Charge Density	mol/kg	2.85
pH stability range	pH	1–13
Thickness (dry)	µm	120

**Table 2:** Summary of the experimental tests carried out in the present work.

Test Number	Test Code	Saline Solution Description	Volume of Brine [L]	[Na^+^] [g/L]	[K^+^] [g/L]	[Mg^2+^] [g/L]	[Ca^2+^] [g/L]	[Cl^-^] [g/L]	[SO_4_^2-^] [g/L]	TDS [g/L]
1	Br-1a	Real industrial brine produced in coal mining activities after a NF step (low concentration)	1	11.27	0.13	0.74	0.70	19.12	2.40	34
2	Br-1b	Real industrial brine for reproducibility purposes	1	10.06	0.14	0.77	0.80	17.72	2.13	32
3	Br-2	Real industrial brine produced in coal mining activities after a NF step (high concentration)	2	10.70	0.13	1.13	1.12	19.85	2.77	36
4	Br-3	Artificial solution mimicking a mining brine enriched in magnesium ions	1	10.07	0.14	3.20	0.80	24.84	2.13	41
5	SW-1a	Real seawater solution sampled from north coast of Sicily	2	11.69	0.41	1.47	0.49	20.85	2.86	38
6	SW-1b	Real seawater solution for reproducibility purposes	2	11.69	0.41	1.47	0.49	20.85	2.86	38

## Abbreviations

AEM	anion exchange membrane
CrIEM	Crystallizer with Ion Exchange Membrane
ED	Electrodialysis
IC	Ion Chromatography
IEM	Ion Exchange Membranes
MDC	Membrane Distillation Crystallization
TDS	Total Dissolved Solid
